# Exploring Gender Differences in Veterans in a Secondary Analysis of a Randomized Controlled Trial of Mindfulness for Chronic Pain

**DOI:** 10.1089/whr.2023.0086

**Published:** 2024-02-12

**Authors:** Diana J. Burgess, Emily M. Hagel Campbell, Mariah Branson, Collin Calvert, Roni Evans, Kelli D. Allen, Ann Bangerter, Lee J.S. Cross, Mary A. Driscoll, Sierra Hennessy, John E. Ferguson, Jessica K. Friedman, Marianne S. Matthias, Laura A. Meis, Melissa A. Polusny, Stephanie L. Taylor, Brent C. Taylor

**Affiliations:** ^1^ VA HSR&D Center for Care Delivery and Outcomes Research, Minneapolis VA Health Care System, Minneapolis, Minnesota, USA.; ^2^ Department of Medicine, University of Minnesota Medical School, Minneapolis, Minnesota, USA.; ^3^ Integrative Health and Wellbeing Research Program, Center for Spirituality and Healing, School of Nursing, University of Minnesota, Minneapolis, Minnesota, USA.; ^4^ VA HSR&D Center of Innovation to Accelerate Discovery and Practice Transformation, Durham VA Health Care System, Durham, North Carolina, USA.; ^5^ Thurston Arthritis Research Center, University of North Carolina at Chapel Hill, North Carolina, USA.; ^6^ Pain Research, Informatics, Multimorbidities, and Education (PRIME) Center, VA Connecticut Health Care System, West Haven, Connecticut, USA.; ^7^ Department of Psychiatry, Yale School of Medicine, New Haven, Connecticut, USA.; ^8^ VA HSR&D Center for the Study of Health Care Innovation, Implementation and Policy, Greater Los Angeles VA Health Care System, Los Angeles, California, USA.; ^9^ VA HSR&D Center for Health Information and Communication, Roudebush VA Medical Center, Indianapolis, Indiana, USA.; ^10^ Regenstrief Institute, Indianapolis, Indiana, USA.; ^11^ Department of Medicine, School of Medicine, Indiana University, Indianapolis, Indiana, USA.; ^12^ Department of Health Policy and Management, UCLA School of Public Health, Los Angeles, California, USA.; ^13^ Department of Medicine, UCLA School of Medicine, Los Angeles, California, USA.

**Keywords:** chronic pain, mindfulness, veterans, women

## Abstract

**Background:**

Although studies have documented higher rates of chronic pain among women Veterans compared to men Veterans, there remains a lack of comprehensive information about potential contributors to these disparities.

**Materials and Methods:**

This study examined gender differences in chronic pain and its contributors among 419 men and 392 women Veterans, enrolled in a mindfulness trial for chronic pain. We conducted descriptive analyses summarizing distributions of baseline measures, obtained by survey and through the electronic health record. Comparisons between genders were conducted using chi-square tests for categorical variables and *t*-tests for continuous measures.

**Results:**

Compared to men, women Veterans were more likely to have chronic overlapping pain conditions and had higher levels of pain interference and intensity. Women had higher prevalence of psychiatric and sleep disorder diagnoses, greater levels of depression, anxiety, post-traumatic stress disorder, fatigue, sleep disturbance, stress and pain catastrophizing, and lower levels of pain self-efficacy and participation in social roles and activities. However, women were less likely to smoke or have a substance abuse disorder and used more nonpharmacological pain treatment modalities.

**Conclusion:**

Among Veterans seeking treatment for chronic pain, women differed from men in their type of pain, had greater pain intensity and interference, and had greater prevalence and higher levels of many known biopsychosocial contributors to pain. Results point to the need for pain treatment that addresses the comprehensive needs of women Veterans.

Clinical Trial Registration Number: NCT04526158. Patient enrollment began on December 4, 2020.

## Background

Chronic pain is a prevalent, debilitating worldwide problem, which disproportionately affects women.^1–7^ Women are more likely to use analgesics, including opioids, and to seek medical help for their pain.^8^ Women are more likely to develop chronic pain than men and report greater pain intensity, pain-related disability, emotional distress, and poorer emotional and social functioning.^1,3,4,8–18^ Women have been shown to have higher prevalence rates of 45 out of 47 chronic pain conditions^19^ and are more likely to have 2 or more chronic overlapping pain conditions (COPCs), a set of painful chronic conditions with a high degree of co-occurrence, thought to have a shared etiology and often accompanied by fatigue, sleep difficulty, psychosocial vulnerability, and pain amplification.^20,21^

When studying gender- and sex-based disparities in chronic pain, it is important to differentiate between sex, a *biological construct*, based on a cluster of anatomical and physiological traits (sex traits), and gender, a *social construct* that refers to roles, behaviors, and identities.^22^ In the past two decades, there has been an increase in research on sex-dependent biological pain mechanisms, including sex differences in hormones, genetics, nervous system, and immune system functioning.^1,4,8^ The current biopsychosocial approach to understanding chronic pain has also led to greater focus on psychosocial contributors to gender disparities in pain and those that result from or are exacerbated by chronic pain, including their interplay with biological factors. For example, the gendered experiences of early life adversity (*i.e.,* childhood physical or sexual abuse, parental neglect, household dysfunction) and sexual trauma, which are more common among women (a social contributor), can affect biological contributors to pain and the experience of pain.^1,23^

Trauma exposure is a risk factor for sleep disorders such as insomnia (considered a biological contributor),^24^ which women are also more likely to experience,^25,26^ and which play a key role in the development and exacerbation of chronic pain.^27^ Mental health disorders and symptoms (psychological contributors) such as depression, anxiety, and post-traumatic stress disorder (PTSD) are more prevalent among women, due, in part, to gendered experiences of trauma, and are associated with a greater likelihood of experiencing chronic pain and poorer pain outcomes.^4,28^ There is also evidence, although mixed, that gender differences in pain coping strategies may also contribute to gender disparities in pain.

Some studies have found women to be more likely than men to engage in maladaptive coping strategies (*e.g*., catastrophizing) that contribute to and exacerbate pain,^18^ while others have found no gender differences.^29^ Other reviews have concluded that women are more likely to engage in adaptive strategies, such as seeking social and emotional support^4^ and utilizing nonpharmacologic pain treatment.^30,31^

Gender disparities in chronic pain are also problematic among U.S. military Veterans,^32–34^ a population that is more likely to experience elevated rates of chronic pain and mental health comorbidities than civilians.^35^ As in the civilian population, compared to men, women Veterans with chronic pain report pain that is more severe.^34,36^ They are more likely to have multiple and COPCs,^34,37^ co-occurring mental health problems,^10,34^ and other factors that contribute to pain, including experiences of military sexual trauma (MST)^38,39^ and childhood trauma.^38,40^ Women Veterans are also more likely to smoke than men Veterans.^41–43^ In addition, compared to women in the civilian population, women Veterans have greater exposure to trauma and severe sexual abuse as well as higher rates of PTSD.^44^

Despite recent scientific advances, there is a need for more information to inform treatment approaches aimed at reducing gender disparities in chronic pain, including research examining a broad range of potential gender differences in known contributors to and correlates of pain that are amenable to intervention (*e.g*., smoking, alcohol, poor sleep, perceived stress, pain-related cognitions).^7,45^ Within the Veteran population, research to address gender disparities in pain has mainly relied on small, convenience samples of women^32^ and from data obtained from the electronic health record (EHR).^33,34,37^ There is also a lack of evidence among treatment-seeking Veterans, as interventions to reduce chronic pain in Veteran populations generally have small samples of women and outcomes are often not broken down by gender.

The purpose of this article is to explore gender differences in pain and its contributors among U.S. military Veterans, using baseline survey and EHR data from the Learning to Apply Mindfulness to Pain (LAMP) study, and to describe the LAMP study participants. LAMP was conducted Veterans with chronic pain who receive care within the VA Health Care System (VA).

LAMP was designed to address the biopsychosocial needs of women Veterans, including barriers to treatment, and was statistically powered to examine the effects of mindfulness-based interventions (MBIs) on women.^46^

## Materials and Methods

Data for this secondary analysis were obtained from the LAMP study, a three-arm randomized pragmatic type 1 hybrid-effectiveness trial of two approaches to delivering a MBI for chronic pain. The trial was conducted within the Minneapolis, Greater Los Angeles, and Durham VA Health Care Systems. The LAMP study was approved by the VA Central Institutional Review Board before data collection (C-IRB No. 18–21). A full description of the study aims and further details about the intervention and methods can be found in our study protocol publication.^46^

### Inclusion/exclusion criteria

To be included, patients had to have had two qualifying pain diagnoses within the same diagnostic category on at least two occasions, at least 90 days apart, during the previous 2 years, a pain duration of ≥6 months, a pain severity score of ≥4 during the past week on the 0–10 Numeric Rating Scale,^31^ access to a smart phone and internet, and willingness to engage in intervention-specific procedures (*e.g*., to meet remotely online for sessions, to download the study mobile app). Patients were excluded if they (1) had a new diagnosis of schizophrenia, bipolar disorder, or active psychosis within the past 18 months in their EHR, (2) had current psychotic symptoms, suicidality, severe depression, a manic episode, or poorly controlled bipolar disorder based on chart review, or (3) were currently enrolled in another pain study or in a Mindfulness-Based Stress Reduction program.

## Measures

### Sociodemographic characteristics

Gender was defined based on the self-reported measure of gender, with four response options (man, woman, another gender, decline to answer). Information recorded in the legacy “birth-sex” variable was used to impute missing responses. Responses were dichotomized as “woman” or “man” in analysis due to the small number of responses in other categories. EHR data were used to assess age, marital status, rurality, and MST. Survey questions assessed race, ethnicity, household financial situation, education, employment status, and the impact of the coronavirus pandemic.^47^

### Pain and mental and behavioral health diagnoses

Chronic pain and mental health diagnoses were assessed using ICD-10 codes obtained from the VA EHR in past year.^46^ COPCs were assessed using an algorithm developed by Schrepf and colleagues (2020), which comprised the following conditions: temporomandibular disorders, fibromyalgia, irritable bowel syndrome, vulvodynia, myalgic encephalomyelitis/chronic fatigue syndrome, urologic chronic pelvic pain syndrome, endometriosis, chronic tension-type headache, migraine headache, and chronic lower back pain.^20^ We created a code indicating whether patients had at least two of these COPCs. Smoking status used EHR data and was based on a pre-established algorithm.^48^

### Pain and functioning

#### Pain intensity and interference

The brief pain inventory (BPI) subscales were used to assess participant experiences of pain intensity (range, 0–10) and interference (range, 0–10) during the past week.^36,37^ Pain interference was assessed by 7 items asking the extent to which pain interferes with general activity, mood, walking ability, normal work, relationships with other persons, sleep, and enjoyment of life on an 11-point numeric rating scale from “Does not interfere” (0) to “Completely interferes” (10). Pain intensity was assessed by 4 items asking participants to rate their worst, least, average, and current pain severity for the past 1 month on an 11-point numeric rating scale from “No pain” (0) to “Pain as bad as you can imagine” (10). A one-point change in BPI interference or intensity is considered clinically significant.^49–52^

High impact chronic pain (HICP) was assessed by the Graded Chronic Pain Scale-Revised, a 2-item measure that assesses pain duration and impact.^53^ HICP is defined as the presence of pain on at least half of days in the previous 3–6 months with substantial restriction of functional participation in work, social, and self-care activities.

#### Functional outcomes

Physical function, anxiety, fatigue, sleep disturbance, participation in social roles, and activities were assessed by The Patient-Reported Outcomes Measurement Information System-29 v20 (PROMIS) Profile, which uses 4 items per domain assessed on a 1–5 scale.^54,55^

Depression was assessed using the Patient Health Questionnaire depression scale (PHQ-8) to evaluate depressive symptoms (range 0–24).^39^ Participants indicated how often they have been bothered by eight possible symptoms over the past 2 weeks. Each item was rated “Not at all” (0), “Several days” (1), “More than half the days” (2), and “Nearly every day” (3).^56^

PTSD symptoms were assessed by the PTSD Checklist for Diagnostic and Statistical Manual of Mental Disorders, Fifth Edition (PCL5).^57^ The PCL5 is a 20-item self-report measure that assesses the presence and severity of PTSD symptoms over the past month (range, 0–80). Respondents are asked to rate how bothered they have been by each of the 20 items on a 5-point Likert scale. Response options for all items are as follows: “Not at all” (0), “A little bit” (1), “Moderately” (2), “Quite a bit” (3), or “Extremely” (4).

Stress was assessed by the NIH Toolbox Perceived Stress Survey (PSS),^58^ comprised 10 items from the Perceived Stress Scale that assesses how “unpredictable, uncontrollable, and overloading respondents find their lives.”^59^ Respondents respond on a 5-point scale ranging from 0 (never) to 5 (very often) to items such as (“that difficulties were piling up so high that you could not overcome them.”)

Alcohol misuse was assessed by the 3-item Alcohol Use Disorders Identification Test (AUDIT), a brief alcohol use disorder screening instrument that reliably identifies individuals who are at risk for alcohol use disorder (including alcohol abuse or dependence). It has 3 items (each scored from 0–4) asking about the frequency and amount of alcohol use.^60^ We used validated cut points of greater than or equal to three for women and greater than or equal to four for men to classify participants as having a positive screen for alcohol consumption.

Pain Catastrophizing was assessed using the Pain Catastrophizing Scale (PCS).^35^ The PCS is a 13-item instrument that asks respondents to reflect on past painful experiences and indicate their thoughts and feelings in response to pain (range, 0–52). Response options for all items are as follows: “Not at all” (0), “To a slight degree” (1), “To a moderate degree” (2), “To a great degree” (3), or “All the time” (4).^61^

Pain self-efficacy was assessed by the Pain Self-Efficacy Questionnaire (PSEQ), a 10-item instrument that assesses the confidence people have in performing activities while in pain. Items are assessed on a 7-point scale from 0 “Not at all confident” to 6 “Completely confident.”

Mindfulness was assessed by the Applied Mindfulness Process Scale (AMPS),^62^ a 15-item self-report measure that asks how often respondents have used mindfulness (in several different forms, such as observing thoughts in a detached manner) to cope with daily stressors over the past 7 days. Answer options are on a 4-point scale from 0 “Never” to 4 “Almost always,” with higher scores indicating more frequent use of a specific mindfulness practice.

#### Pain treatment

Prescriptions for long-term opioid therapy and benzodiazepines over the past year were obtained from the EHR, using established definitions.^63^ Prior nonpharmacological pain treatment and other pain treatment in the past 3 months were assessed by the Nonpharmacological and Self-Care Approaches Measure from the Pain Management Collaboratory, a 9-item instrument which evaluates multiple aspects of engagement in nonpharmacological pain management approaches.^64^

### Statistical analysis

We conducted descriptive analyses summarizing distributions of the baseline measures of participant demographics, pain and other health diagnoses, and self-reported functioning scores for the whole sample and by gender. For categorical variables, *n'*s and percent were reported while means and standard deviations are reported for continuous variables, Comparisons between genders were conducted using chi-square tests for categorical variables and *t*-tests for continuous measures using a significance level of 0.05. Analyses were completed in SAS version 9.4.

## Results

### Study participants

**FIG. 1.**
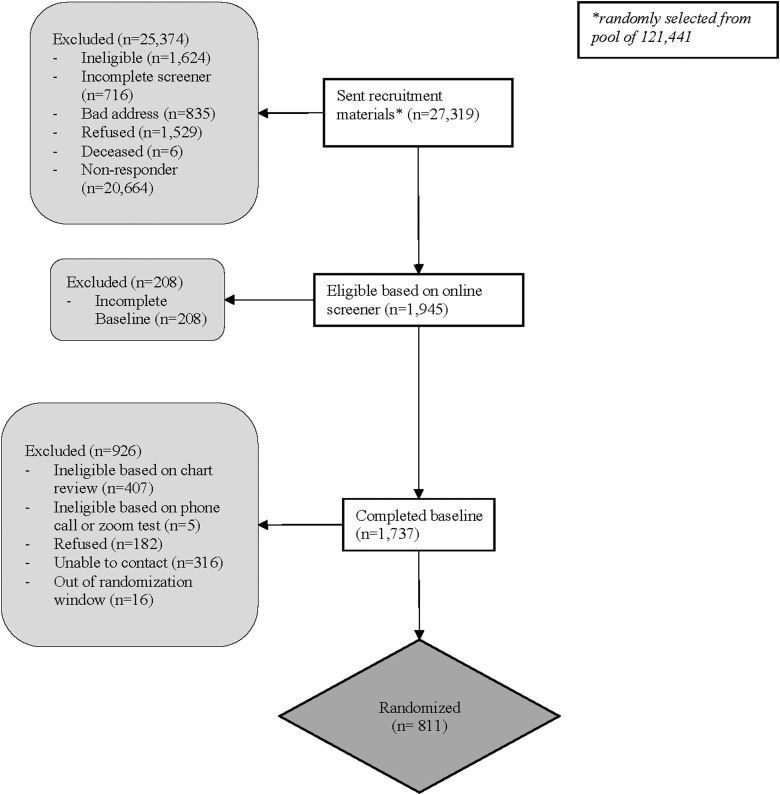
CONSORT flow diagram.

Study participants were recruited from November 2020 to May 2022. Figure 1 delineates participant enrollment and follow-up. Of the 27,319 patients who were sent recruitment materials, 1945 were eligible based on the online screener. One thousand seven hundred thirty-seven of these completed the baseline survey. Nine hundred twenty-six of baseline survey completers were excluded for the following reasons: ineligible based on chart review (*n* = 407), ineligible based on phone call or Zoom test (*n* = 5), refusal (mostly due to inability to meet at scheduled times; *n* = 182), not able to be contacted by phone (*n* = 316), or were outside of the randomization window (*n* = 16). Eight hundred eleven were enrolled in the trial; this comprises the sample for this study.

#### Sociodemographic characteristics

Agreement between gender obtained by survey self-report and the EHR legacy birth-sex variable was 99.1% for those classified as men and 98.5% for those classified as women. Compared to men, women in our sample were younger, more likely to be a member of a minoritized group, less likely to be married, more likely to be employed, and more likely to have a 4-year or advanced degree (Table 1). Women were also more likely to have experienced MST and to report that the coronavirus pandemic adversely affected their mental and emotional health.

**Table 1.** Sociodemographic Characteristics by Gender

	Men	Women	Total	Significance
	424	387	811	
Demographics				
Age, from EHR, Mean (SD), years	58.4 (12.6)	50.5 (11.8)	54.6 (12.9)	<0.0001
Ethnicity, *N* (%), Hispanic/Latino	21 (5.0)	30 (7.8)	51 (6.3)	0.10
Race, *N* (%)				<0.0001
Black or African American	70 (16.6)	138 (35.8)	208 (25.8)	
American Indian/Alaska Native	6 (1.4)	4 (1.0)	10 (1.2)	
Asian American	3 (0.7)	3 (0.8)	6 (0.7)	
Native Hawaiian/Pacific Islander	0 (0)	1 (0.3)	1 (0.1)	
White	327 (77.5)	219 (56.9)	546 (67.7)	
Multiracial	16 (3.8)	20 (5.2)	36 (4.5)	
Household financial situation, *N* (%)				0.09
Live comfortably	134 (31.6)	114 (29.5)	248 (30.6)	
Meet your basic expenses with a little left over for extras	190 (44.8)	154 (39.8)	344 (42.4)	
Just meet your basic expenses	89 (21.0)	100 (25.8)	189 (23.3)	
Don't even have enough to meet basic expenses	11 (2.6)	19 (4.9)	30 (3.7)	
Marital status, from EHR, *N* (%)				<0.0001
Married	276 (65.1)	152 (39.3)	428 (52.8)	
Divorced/separated/widowed	90 (21.2)	135 (34.9)	225 (27.7)	
Never married/single	54 (12.7)	92 (23.8)	146 (18.0)	
Unknown	4 (1.0)	8 (2.1)	12 (1.5)	
Employment status, *N* (%)				<0.0001
Working now	152 (35.9)	181 (46.8)	333 (41.1)	
Disabled	89 (21.0)	88 (22.7)	177 (21.8)	
Retired	142 (33.5)	62 (16.0)	204 (25.2)	
Other	41 (9.7)	56 (14.5)	97 (12.0)	
Education, *N* (%)				0.009
High school or less	36 (8.5)	17 (4.4)	53 (6.5)	
Some college	197 (46.5)	157 (40.6)	354 (43.7)	
Bachelors	109 (25.7)	112 (28.9)	221 (27.3)	
Masters+	82 (19.3)	101 (26.1)	183 (22.6)	
Rurality, from EHR, *N* (%)				0.27
Urban	265 (62.5)	233 (60.2)	498 (61.4)	
Rural	138 (32.6)	127 (32.8)	265 (32.7)	
Highly rural	6 (1.4)	3 (0.8)	9 (1.1)	
Military sexual trauma, from EHR	22 (5.2)	148 (38.4)	170 (21.1)	<0.0001
Impact of coronavirus pandemic				
Ability to get health care	201 (47.4)	162 (41.9)	363 (44.8)	0.11
Social support	253 (59.7)	221 (57.1)	474 (58.5)	0.46
Finances	162 (38.2)	173 (44.7)	335 (41.3)	0.06
Ability to meet basic needs	133 (31.4)	133 (34.4)	266 (32.8)	0.36
Mental and emotional health	288 (68.0)	296 (76.5)	584 (72.0)	0.007
Site, from her				<0.0001
Durham	149 (35.1)	193 (49.9)	342 (42.2)	
Minneapolis	218 (51.4)	127 (32.8)	345 (42.5)	
Los Angeles	57 (13.4)	67 (17.3)	124 (15.3)	

Unless specified, all variables were obtained by self-report.

EHR, electronic health record; SD, standard deviation.

#### Pain diagnoses

Compared to men, women were more likely to have a number of pain diagnoses, including fibromyalgia and wide-spread muscle pain, headache, abdominal and bowel pain, orofacial, ear, and temporomandibular disorder pain, neck pain, urogenital pain, systemic disorders or diseases causing pain, and COPCs. The only pain diagnosis women were less likely to have than men was neuropathy (Table 2).

**Table 2.** Pain Diagnoses by Gender

	Man	Woman	Total	Significance
	424	387	811	
At least two COPCs	33 (7.8)	79 (20.4)	112 (13.8)	<0.0001
Number of COPCs among patients with COPCs^a^ Mean (SD)	2.15 (0.36)	2.37 (0.56)	2.30 (0.52)	0.02
Abdominal and bowel pain	59 (13.9)	73 (18.9)	132 (16.3)	0.06
Back pain	210 (49.5)	177 (45.7)	387 (47.7)	0.28
Bone infections	5 (1.2)	3 (0.8)	8 (1.0)	0.56
Fibromyalgia and wide-spread muscle pain	92 (21.7)	127 (32.8)	219 (27.0)	0.0004
Fractures, contusions, sprains, and strains	47 (11.1)	43 (11.1)	90 (11.1)	0.99
Headache	50 (11.8)	85 (22.0)	135 (16.7)	0.0001
Infectious arthritic diseases	6 (1.4)	2 (0.5)	8 (1.0)	0.20
Limb extremity pain, joint pain, and arthritic disorders	288 (67.9)	274 (70.8)	562 (69.3)	0.38
Musculoskeletal chest pain	36 (8.5)	23 (5.9)	59 (7.3)	0.16
Neck pain	99 (23.4)	114 (29.5)	213 (26.3)	0.05
Neuropathy	64 (15.1)	32 (8.3)	96 (11.8)	0.003
Orofacial, ear, and temporomandibular disorder pain	5 (1.2)	13 (3.4)	18 (2.2)	0.04
Urogenital pain	12 (2.8)	47 (12.1)	59 (7.3)	<0.0001
Other painful conditions	54 (12.7)	55 (14.2)	109 (13.4)	0.54
Systemic disorders or diseases causing pain	12 (2.8)	24 (6.2)	36 (4.4)	0.02

All diagnoses obtained from the EHR. *N* (%) except when indicated.

^a^ COPCs were composed of the following conditions: temporomandibular disorders, fibromyalgia, irritable bowel syndrome, vulvodynia, myalgic encephalomyelitis/chronic fatigue syndrome, urologic chronic pelvic pain syndrome, endometriosis, chronic tension-type headache, migraine headache, and chronic lower back pain

COPCs, chronic overlapping pain conditions.

#### Mental and behavioral health diagnoses

Except for substance use disorder, which was higher among men, women were more likely than men to be diagnosed with each of the mental health conditions coded, including depression, anxiety, and PTSD, and were more likely to have been diagnosed with a sleep disorder (Table 3).

**Table 3.** Mental and Behavioral Health Diagnoses by Gender, *N* (%)

	Man	Woman	Total	Significance
	424	387	811	
Any mental health diagnosis	229 (54.0)	280 (72.4)	509 (62.8)	<0.0001
Depressive disorders	133 (31.4)	191 (49.4)	324 (40.0)	<0.0001
Anxiety disorders	82 (19.3)	117 (30.2)	199 (24.5)	0.0003
PTSD	84 (19.8)	122 (31.5)	206 (25.4)	0.0001
Opioid use disorder	1 (0.2)	0 (0.0)	1 (0.1)	0.34
Substance use disorders	42 (9.9)	19 (4.9)	61 (7.5)	0.007
Sleep diagnoses	50 (11.8)	78 (20.2)	128 (15.8)	0.001
Nicotine dependence	24 (5.7)	26 (6.7)	50 (6.2)	0.53

All diagnoses obtained from the EHR.

PTSD, post-traumatic stress disorder.

#### Pain and functioning

Women reported worse pain and functioning across all domains assessed, except alcohol use, mindfulness skills, and HICP, for which no differences were found. Specifically, women reported higher levels of pain interference, pain intensity, depression, anxiety, PTSD symptoms, fatigue, sleep disturbance and stress, greater pain catastrophizing and lower levels of pain self-efficacy and participation in social roles and activities, and were less likely to be a current smoker (Table 4).

**Table 4.** Pain and Functioning by Gender

	Man	Woman	Total	Significance
	424	392	811	
BPI—interference	5.38 (1.89)	5.82 (2.02)	5.59 (1.97)	0.0014
BPI—pain severity (intensity)	5.35 (1.48)	5.73 (1.60)	5.53 (1.55)	0.0006
HICP (Graded chronic pain scale-revised-Revised)	0.63 (0.48)	0.63 (0.48)	0.63 (0.48)	0.94
Physical function (PROMIS)	12.64 (3.51)	12.00 (3.19)	12.33 (3.37)	0.007
Anxiety (PROMIS)	9.27 (3.73)	10.41 (3.95)	9.81 (3.88)	<0.0001
Fatigue (PROMIS)	12.95 (3.87)	14.52 (4.09)	13.70 (4.05)	<0.0001
Sleep disturbance (PROMIS)	13.69 (3.71)	14.70 (3.57)	14.17 (3.68)	<0.0001
Participation in social roles and activities (PROMIS)	10.51 (3.18)	10.11 (3.10)	10.32 (3.15)	0.07
PHQ-8 depression symptoms	8.88 (5.74)	10.29 (5.69)	9.55 (5.75)	0.0005
PTSD (PCL5)	25.94 (19.25)	28.65 (19.63)	27.24 (19.47)	0.05
Unhealthy alcohol use (AUDIT-C)	3.07 (2.11)	2.30 (1.86)	2.70 (2.03)	<0.0001
AUDIT-C positive screen, *N* (%)	90 (32.0)	81 (31.2)	171 (31.6)	0.83
Smoking status, *N* (%)- obtained from her				<0.0001
Current smoker	51 (12.0)	38 (9.8)	89 (11.0)	
Former smoker	195 (46.0)	110 (28.4)	305 (37.6)	
Never smoker	170 (40.1)	224 (57.9)	394 (48.6)	
Unknown	8 (1.9)	15 (3.9)	23 (2.8)	
PCS	23.64 (11.07)	21.24 (11.34)	22.40 (11.27)	0.003
PSEQ	30.57 (12.28)	32.35 (11.91)	31.49 (12.11)	0.08
Perceived stress fixed form	31.74 (3.99)	30.89 (4.08)	31.30 (4.05)	0.002
Applied mindfulness process scale	26.06 (13.74)	26.46 (14.05)	26.27 (13.89)	0.64

Unless specified, all variables were obtained by self-report. Mean (SD) except when indicated.

AUDIT-C, alcohol use disorders identification test-concise; BPI, brief pain inventory; HICP, high impact chronic pain; PCL5, 5-item PTSD checklist; PCS, Pain Catastrophizing Scale; PHQ-8, 8-item Patient Health Questionnaire; PROMIS, Patient-Reported Outcomes Measurement Information System); PSEQ, Pain Self-Efficacy Questionnaire.

#### Pain treatment

Women were more likely to report using manipulation or chiropractic care, therapeutic massage, yoga, relaxation techniques, mindfulness, psychotherapy, and topical pain relievers (Table 5). There were no differences in the use of Tai Chi/Qigong, exercise, acupuncture and in the use of opioids, benzodiazepines, spinal injections, and nonopioid medications.

**Table 5.** Pain Treatment by Gender, *N* (%)

	Man	Women	Total	Significance
	424	387	811	
Prescription from her				
Long-term opioid therapy	101 (23.8)	91 (23.5)	192 (23.7)	0.92
Benzodiazepine	33 (7.8)	35 (9.0)	68 (8.4)	0.52
Long-term opioid and benzodiazepine	17 (4.0)	11 (2.8)	28 (3.5)	0.36
Prior nonpharmacological pain treatment in past 3 months				
Acupuncture	65 (15.3)	77 (19.9)	142 (17.5)	0.09
Manipulation	132 (31.1)	150 (38.8)	282 (34.8)	0.02
Massage	148 (34.9)	189 (48.8)	337 (41.6)	<0.0001
Yoga	61 (14.4)	101 (26.1)	162 (20.0)	<0.0001
Tai chi/qigong	25 (5.9)	20 (5.2)	45 (5.6)	0.65
Exercise	299 (70.5)	273 (70.5)	572 (70.5)	0.99
Relaxation techniques	163 (38.4)	205 (53.0)	368 (45.4)	<0.0001
Meditation/mindfulness	102 (24.1)	121 (31.3)	223 (27.5)	0.02
Psychotherapy/counseling	86 (20.3)	120 (31.0)	206 (25.4)	0.0005
Other pain treatment in past 3 months				
Spinal injections	45 (10.6)	45 (11.6)	90 (11.1)	0.65
Opioid medications used for pain	108 (25.5)	87 (22.5)	195 (24.0)	0.32
Nonopioid medications used for pain	346 (81.6)	325 (84.0)	671 (82.7)	0.37
Topical pain relievers	310 (73.1)	316 (81.7)	626 (77.2)	0.004

Unless specified, all variables were obtained by self-report.

## Discussion

This study advances our understanding about gender disparities among Veterans seeking treatment for chronic pain and identifies potential ways in which treatment approaches may be better tailored to women. Many of these gender difference replicate those found in the civilian population. Women Veterans in our sample reported greater pain intensity and interference and were more likely to have a number of biopsychosocial factors known to have complex and intersecting relationships to pain. This includes poorer emotional and social functioning, higher levels of perceived stress and fatigue, greater likelihood of having a mental health disorder or sleep disorder, higher levels of pain catastrophizing, lower levels of pain self-efficacy and higher levels of trauma (MST).

However, women had several protective factors. They were more likely to use several complementary and integrative pain treatment approaches (acupuncture, manipulation/chiropractic, massage, yoga) and were less likely to have a substance abuse disorder or to smoke. In contrast to prior findings,^65^ women Veterans were no more likely to have higher rates of opioid medications and benzodiazepines than men (separately and coprescribed).

Compared to their men, women Veterans were more likely to have COPCs and pain conditions that have recently been described as nocioplastic—chronic pain resulting from the abnormal processing of pain signals without clear evidence of tissue damage or somatosensory system pathology (previously described as functional pain syndromes),^66,67^ such as fibromyalgia and widespread muscle pain. While these gender differences were similar to those found in civilian^1,4,8^ and Veteran populations,^10,34,37^ the prevalence of some of the diagnoses in this treatment-seeking sample far exceeds what has been reported in epidemiological studies.^34,37^ For example, 21.7% of men and 32.8% of women in our sample had a diagnosis of fibromyalgia.

A major strength of this study was our ability to obtain survey and electronic health data from a large, relatively balanced sample of men and women with chronic pain (including over 40% women from minoritized groups), which enabled us to examine gender differences in a wide range of pain and its contributors, grounded in a biopsychosocial model. However, there are several limitations that future studies should address. First, we did not assess participants' sexual orientation.^68^ We also did not assess potentially important contributors to gender differences in pain, such as early life adversity, lifetime trauma, and experiences of discrimination.^4^

Finally, our sample differs from the larger population of people with chronic pain in important ways. For example, consistent with other studies of the VA population, compared to their men, women in our sample were more likely to belong to a minoritized group, which has been associated with more severe pain,^6^ but also are more likely to be younger, to be employed, and to have higher levels of education, all of which have been negatively associated with pain.^6,7^ We did not adjust for these factors in our comparisons, as our study was intended to generalize to the population of men and women Veterans with chronic pain.

These findings have clinical and policy implications for the many women Veterans affected by chronic pain. First, women Veterans may benefit from treatment approaches that address the specific psychological (*e.g*., pain catastrophizing and comorbid mental health conditions), biological (*e.g*., insomnia and other sleep disorders), and social (*e.g*., exposure to trauma, interference in social activities) contributors to pain that disproportionately affects them. These approaches may include evidence-based psychological treatments for pain (*e.g*., MBIs, cognitive behavioral therapy for pain),^69^ multimodal approaches that include complementary and integrative approaches that have been found to be appealing to women Veterans (*e.g*., acupuncture, chiropractic, massage, yoga) and programs adapted to be trauma-informed. Likewise, efforts to augment existing evidence-based psychological interventions to address co-occurring concerns (*e.g*., trauma/pain), and to explicitly target social functioning, may be warranted.

It is also important that women Veterans have access to recommended treatment strategies for nocioplastic pain syndromes, which prioritize nonpharmacologic approaches and which focus on reducing, versus eradicating, symptoms and improving function.^67^ In addition, because women are more likely to experience stigmatization, due to their gender and also to the type of pain they disproportionately experience (*e.g*., headache, overlapping pain conditions, fibromyalgia),^70,71^ it is critical that women are able to access providers with sufficient knowledge and training in the biopsychosocial model of pain, including in how to communicate effectively and sensitively. The importance of addressing and integrating COPCs into conceptualizations and treatment plans also will be important to ensure optimal outcomes.

As women Veterans are more likely to be younger and employed, it is important that care be delivered in ways that accommodate the demands of work and caretaking, such as programs delivered by telemedicine and outside of work hours. Telemedicine also may be desired by women Veterans, whose past experiences of MST may contribute to the avoidance of care in VA.

## Conclusion

Among Veterans seeking treatment for chronic pain, women differed in their type of pain, had greater pain intensity and interference, and had greater prevalence and higher levels of many known biopsychosocial contributors to pain. Results point to the need for treatment approaches designed to address these gender-specific needs in the Veteran population.

## Abbreviations Used

AUDIT-C: alcohol use disorders identification test-concise
BPI: brief pain inventory
COPCs: chronic overlapping pain conditions
DSM: Diagnostic and Statistical Manual of Mental Disorders, Fifth Edition
EHR: electronic health record
HICP: high impact chronic pain
LAMP: learning to apply mindfulness to pain
MBIs: mindfulness-based interventions
MST: military sexual trauma
PCS: Pain Catastrophizing Scale
PHQ-8: Patient Health Questionnaire Depression Scale
PCL5: PTSD checklist for DSM-5
PROMISE: Patient-Reported Outcomes Measurement Information System
PTSD: post-traumatic stress disorder
SD: standard deviation
VA: Veterans Health Administration
